# Small molecule-induced simultaneous destabilization of β-catenin and RAS is an effective molecular strategy to suppress stemness of colorectal cancer cells

**DOI:** 10.1186/s12964-020-0519-z

**Published:** 2020-03-06

**Authors:** Yong-Hee Cho, Eun Ji Ro, Jeong-Su Yoon, Dong-Kyu Kwak, Jaebeom Cho, Dong Woo Kang, Ho-Young Lee, Kang-Yell Choi

**Affiliations:** 1 Translational Research Center for Protein Function Control, Yonsei University, Seoul, South Korea; 2 Department of Biotechnology, College of Life Science and Biotechnology, Yonsei University, Seoul, South Korea; 3 College of Pharmacy and Research Institute of Pharmaceutical Sciences, Seoul National University, Seoul, South Korea; 4 Medpacto Inc., Borim building, 92 myeongdal Ro, Seocho-gu, Seoul, South Korea; 5 CK Biotechnology Inc, Building 117, 50 Yonsei Ro, Seodaemun-Gu, Seoul, South Korea

**Keywords:** Cancer stem cells, β-Catenin, RAS, Destabilization, KYA1797K

## Abstract

**Background:**

Cancer stem cells (CSCs), the major driver of tumorigenesis, is a sub-population of tumor cells responsible for poor clinical outcomes. However, molecular mechanism to identify targets for controlling CSCs is poorly understood.

**Methods:**

Gene Set Enrichment Analyses (GSEA) of Wnt/β-catenin and RAS signaling pathways in stem-like subtype of colorectal cancer (CRC) patients were performed using two gene expression data set. The therapeutic effects of destabilization of β-catenin and RAS were tested by treatment of small molecule KYA1797K using CRC patient derived cells.

**Results:**

Treatment with KYA1797K, a small molecule that destabilizes both β-catenin and RAS via Axin binding, effectively suppresses the stemness of CSCs as shown in CRC spheroids and small intestinal tumors of *Apc*^*Min/+*^*/K-Ras*^*G12D*^*LA2* mice. Moreover, KYA1797K also suppresses the stemness of cells in CRC patient avatar model systems, such as patient-derived tumor organoids (PDTOs) and patient-derived tumor xenograft (PDTX).

**Conclusion:**

Our results suggest that destabilization of both β-catenin and RAS is a potential therapeutic strategy for controlling stemness of CRC cells.

Video abstract

## Background

Colorectal cancer (CRC) is the third most commonly diagnosed cancer worldwide [1], and has remained a major cause of cancer-associated mortality mainly due to the metastasis and recurrence [2]. Cancer stem cells (CSCs), the sub-population of tumor cells that possess stem cell properties, plays critical roles in the initiation and progression of CRC, and are considered as main culprits that contribute to the metastasis and recurrence of CRC [3–6]. A recent clinical study revealed that CRC patients who possess the tumors with high stem cell properties have critically short disease free survival (DFS) rates compared with those who possess differentiated tumors [7]. However, drugs to control CSCs as well as the molecular targets for development of such drugs have not yet been identified.

The Wnt/β-catenin pathway is a major signaling pathway that maintains intestinal homeostasis via regulation of intestinal stem cells (ISCs) and their precursor cells [8]. Mutation of *Adenomatous polyposis coli* (*APC)* which occurs in more than 90% of CRC patients, is an initial event for induction of CSCs and tumor formation. Indeed, convincing molecular markers representing CSCs in CRC including *leucine-rich repeat-containing G-protein coupled receptor 5* (*LGR5*)*, CD44*, and *CD133* are the transcriptional targets of Wnt/β-catenin pathway [9–11]. Recent studies using lineage tracing in animal models revealed that stem-cell-specific loss of *Apc* resulted in progressive growth of neoplasia by transformation of their progenitor cells [3].

*KRAS* mutations, which occur in 50% of advanced CRC patients, activate both extracellular signal-regulated kinase (ERK) and phosphatidyl inositol 3-kinase (PI3K)-AKT signaling pathways, and subsequently induce proliferation of ISCs [12, 13]. Although *Kras* mutation alone does not induce CSC activation or the tumor formation in murine small intestine, co-occurrence of mutations in both *Apc* and *Kras* enriches and activates CSCs via synergistic activation of the Wnt/β-catenin pathway [14–16]. Therefore, inhibition of both pathways has been suggested as an effective strategy for suppression of CSCs as a treatment for CRC [17, 18].

In this study, we analyzed the genomic expression profiles of stem-like subtypes of CRC patient tissues possessing CSC characteristics using two gene expression data set (GSE13294; *N* = 125, GSE14333; *N* = 246) derived from resected primary CRC tissues [7]. Interestingly, we found that the expression levels of the Wnt/β-catenin and RAS/MAPK signaling pathways target genes are highly increased in stem-like subtype of CRC patient tissues compared with other differentiated subtypes of CRC patients. In addition, both β-catenin and RAS protein, as well as the GTP-bound active RAS levels were also highly elevated in the CSC-like populations possessing stem cell characteristics compared with non-CSC like populations of CRC cell lines classified by fluorescent activating cell sorting (FACS). KYA1797K, a small molecule that destabilize both β-catenin and RAS by activating GSK3β via binding with Axin [19], effectively suppressed the stemness of CRC spheroids with their growth inhibition.

Moreover, KYA1797K effectively suppressed the stemness of CSCs in small intestinal tumors of *Apc*^*Min/+*^*/Kras*^*G12D*^*LA2* mice as well as patient avatar models such as patient-derived tumor organoids (PDTOs) and patient-derived tumor xenograft model (PDTX). In addition, KYA1797K effectively induced differentiation in metastatic CRC PDTX tissues as shown by increased expression of KRT20, a convincing differentiation marker of CRC. Taken together, we suggest that a small molecule-induced destabilization of both β-catenin and RAS is the potential therapeutic approach to suppress the stemness of CSCs with clinical implications for treatment of CRC.

## Methods

### Cell cultures and reagents

Human CRC cells (HCT15, DLD1, SW48, SW480, SW620, Widr, RKO, and HCT116) were purchased from the American Type Culture Collection (Manassas, VA, USA). DLD-1 harboring mutant *KRAS* [20]. was provided by B. Vogelstein (John Hopkins Oncology Center, Baltimore, MD, USA). All cell lines were authenticated using short tandem repeat profiling (Cosmogenetech, Korea) and were maintained in RPMI1640 (Gibco, Carlsbad, CA, USA) or DMEM (Gibco) supplemented with 10% fetal bovine serum (Gibco). KYA1797K was dissolved in dimethyl sulfoxide (Sigma-Aldrich, St. Louis, MO, USA) for in vitro studies.

### Immunoblotting

Cells were washed with ice-cold PBS and lysed using radio-immunoprecipitation assay (RIPA) buffer (150 mM NaCl; 10 mM Tris, pH 7.2; 0.1% sodium dodecyl sulfate; 1% Triton X-100; 1% sodium deoxycholate; and 5 mM ethylenediaminetetraacetic acid). Samples of mouse tissues stored in liquid nitrogen were prepared in RIPA buffer and then homogenized. Proteins were separated on a 10–12% sodium dodecyl sulfate polyacrylamide gel and transferred to a nitrocellulose membrane (Whatman, Little Chalfont, UK). Immunoblotting was performed with the following primary antibodies: anti-pan-RAS monoclonal (Millipore, MA, USA, MBS195; 1:3000), anti-β-catenin (Santa Cruz Biotechnology, TX, USA, sc-7199; 1:3000), anti-c-Myc (Santa Cruz Biotechnology, sc-789; 1:1000), anti-p-ERK (Cell Signaling Technology, sc514302; 1:1000), anti-p-AKT (Cell Signaling Technology, #4060 s; 1:1000), and anti- β-actin (Santa Cruz Biotechnology, sc-47,778; 1:5000). Horseradish peroxidase-conjugated anti-mouse (Cell Signaling Technology, #7076; 1:3000) or anti-rabbit (Bio-Rad, Hercules, CA, USA, #1706515; 1:3000) secondary antibodies were used.

### Reverse transcription and quantitative real-time PCR

Total RNA was isolated using Trizol reagent (Invitrogen, Waltham, MA, USA) according to the manufacturer’s instructions. Total RNA (2 μg) was reverse transcribed using 200 U of M-MLV reverse transcriptase (Invitrogen) in a 20-μl reaction mixture at 42 °C for 1 h. For quantitative real-time PCR analyses, the resulting cDNA (1 μl) was amplified in 10 μl of Rotor-gene SYBR green (Bio-Rad). The comparative cycle-threshold (C_T_) method was used, and *β-actin* served as an endogenous control.

### Animal studies

All animal experiments were performed in accordance with the guidelines of the Korean Food and Drug Administration. Protocols were reviewed and approved by the Institutional Animal Care and Use Committee of Yonsei University. The C57BL/6 J-*Apc*^*Min/+*^ (*Apc*^*Min/+*^) and B6.129S-*Kras*^*tm3Tyj*^ mice were obtained from Jackson Laboratory (Bar Harbor, ME, USA) To generate *Apc*^*Min/+*^*/Kras*^*G12D*^*LA2* mice, *Apc*^*Min/+*^ were crossed with *Kras*^*G12D*^*LA2* mice. Mouse genotyping was performed using genomic DNA extracted from the tail. To control for genetic background effects, sex-matched littermates were used as controls. To investigate the in vivo efficacy of KYA1797K, 5-week-old *Apc*^*Min/+*^*/Kras*^*G12D*^*LA2* (*N* = 4) mice were intraperitoneally injected with KYA1797K (25 mg/kg) 5 days per week for 7 weeks. Immediately after sacrifice, the abdomen of each mouse was cut open longitudinally and cleaned by flushing with PBS. The tumors were classified according to standard World Health Organization histopathological criteria. Tumor sizes were classified based on the diameter (small ≤1 mm, 1 mm < medium ≤3 mm, and large > 3 mm). For biochemical analyses, a subset of freshly isolated tissues was snap frozen in liquid nitrogen and stored at − 80 °C.

For the PDTX studies, athymic *nu/nu* mice were injected subcutaneously in the dorsal flank with DLD-*KRAS-*MT (5 × 10^6^ cells/mice) in 200 μl of PBS/Matrigel (BD Biosciences, Bedford, MA, USA; 1:1). When the mean tumor size reached between 100 and 200 mm^3^, the mice were randomly divided into two groups to receive either vehicle or KYA1797K. Twenty-eight days after the initial drug treatment, the mice were sacrificed, and tumors were excised and fixed in 10% neutralized formaldehyde for further analyses.

### Immunohistochemistry

For IHC analyses, 4-μm paraffin-embedded tissue sections were treated with citrate buffer (pH 6.0) and autoclaved for 15 min. The sections were then blocked with 5% bovine serum albumin (BSA; Affymetrix, Santa Clara, CA, USA) and 1% normal goat serum (NGS, Vector Laboratories, Burlingame, CA, USA) in PBS for 30 min. After blocking, sections were incubated with primary antibody overnight at 4 °C followed by incubation with anti-mouse Alexa Fluor 488- (Life Technologies, Carlsbad, CA, USA, A11008; 1:500) or anti-rabbit Alex Fluor 555-conjugated (Life Technologies, A21428; 1:500) secondary antibodies for 1 h at room temperature. The sections were then counterstained with 4′, 6-diamidino-2-phenylindole (DAPI; Sigma-Aldrich) and mounted in Gel/Mount media (Biomeda Corporation, Foster City, CA, USA). All incubations were conducted in dark, humid chambers. The fluorescence signal was visualized using a confocal microscope (LSM510; Carl Zeiss) at excitation wavelengths of 488 nm (Alexa Fluor 488), 543 nm (Alexa Fluor 555), and 405 nm (DAPI). At least three fields per section were analyzed.

### Immunocytochemistry

The cells were fixed with 5% formalin for 30 min, permeabilized with 0.2% Triton X-100 for 30 min, and pre-blocked with PBS containing 5% BSA and 1% NGS for 1 h. The cells were then incubated with the indicated primary antibody overnight at 4 °C, followed by incubation with Alexa Fluor 488- or Alex Fluor 555-conjugated secondary antibodies for 4 h at 4 °C. Samples were then counterstained with DAPI for 10 min at room temperature. After incubation, the cells were mounted in Gel/Mount media (Biomeda Corporation). The fluorescence signal was visualized using a confocal microscope (Zeiss, LSM510) at excitation wavelengths of 488 nm (Alexa Fluor 488), 543 nm (Alexa Fluor 555), and 405 nm (DAPI).

### RAS activation assay and immunoblotting

GTP-loaded active RAS was analyzed by manufacture’s procedure (Cell signaling technology, #11860). Cells were lysed by lysis buffer plus 1 mM PMSF, and cleared by centrifugation at 16,000 x g, for 15 min at 4 °C. 500 μg of cell lysates was incubated with beads coated with fusion protein (GST-Raf1-RBD) at 4 °C for 1 h. Beads were washed 3 times with cold lysis buffer, and bounded protein was eluted with 2x SDS sample buffer, incubated at 100 °C for 5 min and analyzed by immunoblotting for RAS.

### Tumor organoid experiments

For human PDC-derived tumor organoids, 250 cells per 25 μl of growth factor-reduced Matrigel (BD Bioscience) were mixed, and N2 medium containing 10% R-spondin-1 CM, 100 μg/ml noggin (Peprotech, Rocky Hill, NJ, USA), 1.25 mM N-acetyl cysteine (Sigma-Aldrich), 10 mM nicotinamide (Sigma-Aldrich), 50 ng/ml EGF (Peprotech), 10 nM gastrin (Sigma-Aldrich), 500 nM A83–01 (Sigma-Aldrich), and 3 μM SB202190 (Sigma-Aldrich) were added. The growth medium was refreshed every 2 days, and the cells were passaged by mechanical disruption every 10–14 days at a 1:5 ratios. The measurements of tumor organoid growth were performed using the CellTiter-Glo Luminescent Cell Viability Assay (Promega, Madison, WI, USA) according to the manufacturer’s instructions. Luminescence was measured with a FLUOstar Optima instrument (BMG Labtech, Ortenberg, Germany).

For mouse tumor organoid experiments, small intestinal tumors of *Apc*^*Min/+*^*/Kras*^*G12D*^*LA2* mice were isolated and washed with ice-cold PBS, and single cells isolated from tumors were collected using 0.25% trypsin containing 10 μM Ly27632 (Sigma-Aldrich) and 100 μg/ml Primocin (Invivogen, San Diego, CA, USA) for 30 min. After incubation, 1X B27 (Sigma-Aldrich) was added, and the mixture was filtered through 100-μm and 40-μm cell strainers (BD Biosciences) to collect single cells. The cells were then mixed with growth factor-reduced Matrigel. The growth medium was refreshed every 2 days and the cells were passaged by mechanical disruption every 10–14 days at a 1:5 ratios. The measurements of tumor organoid growth were performed using the CellTiter-Glo Luminescent Cell Viability Assay according to the manufacturer’s instructions, and luminescence was measured with a FLUOstar Optima instrument.

### Primary cell culture from human CRC cells and PDTX

CRC PDCs that had previously been established [21] were used after receiving patient-informed consent and approval from the Institutional Review Board of Asan Medical Center. All procedures performed in studies involving human participants were conducted in accordance with the International Ethical Guidelines for Biomedical Research Involving Human Subjects. For the PDTX model, animal procedures were performed following protocols approved by the Seoul National University Institutional Animal Care and Use Committee. PDTX experiments were performed as previously described [22]. Briefly, small pieces of CRC tumors that had metastasized to lung were subcutaneously transplanted into the flanks of 5-week-old NOD/SCID mice (*N* = 5). After the tumor volumes reached 50–150 mm^3^, erlotinib was injected by oral gavage at a dosage of 25 mg/kg 5 days per week for 28 days. KYA1797K was intraperitoneally injected into mice (*N* = 5) at dosage of 25 mg/kg every day for 28 days. Tumor growth was determined by measurement of the short and long diameters of the tumor with a caliper, and tumor volumes were determined according to the following formula: tumor volume (mm^3^) = (short diameter mm) × (long diameter mm) × 0.5.

### Fluorescence activating cell sorting (FACS) analyses

The sorting methods are described in our previous study [23]. Briefly, CRC cell lines (SW480, HCT116, DLD-1, SW620, SW48, and HT29) were dissociated into single cells by trypsin-EDTA, incubated with anti-human CD133-PE (Miltenyi Biotec, Bergisch Gladach, Germany), anti-human CD166-PerCP-eFluor (eBioscience, Waltham, MA, USA), and anti-human/mouse CD44-APC (eBioscience), and sorted by FACS (BD Bioscience).

### Statistical analysis

All data are represented as the mean ± standard deviation of at least three independent experiments. The statistical significance of differences was assessed using the Student’s *t-*test. Significance was denoted as *n.s.* not significant, ^*^P < 0.05, ^**^P < 0.01, and ^***^P < 0.001.

## Results

### Both Wnt/β-catenin and RAS signaling pathways are activated in CRC harboring CSC properties

**Fig. 1:**
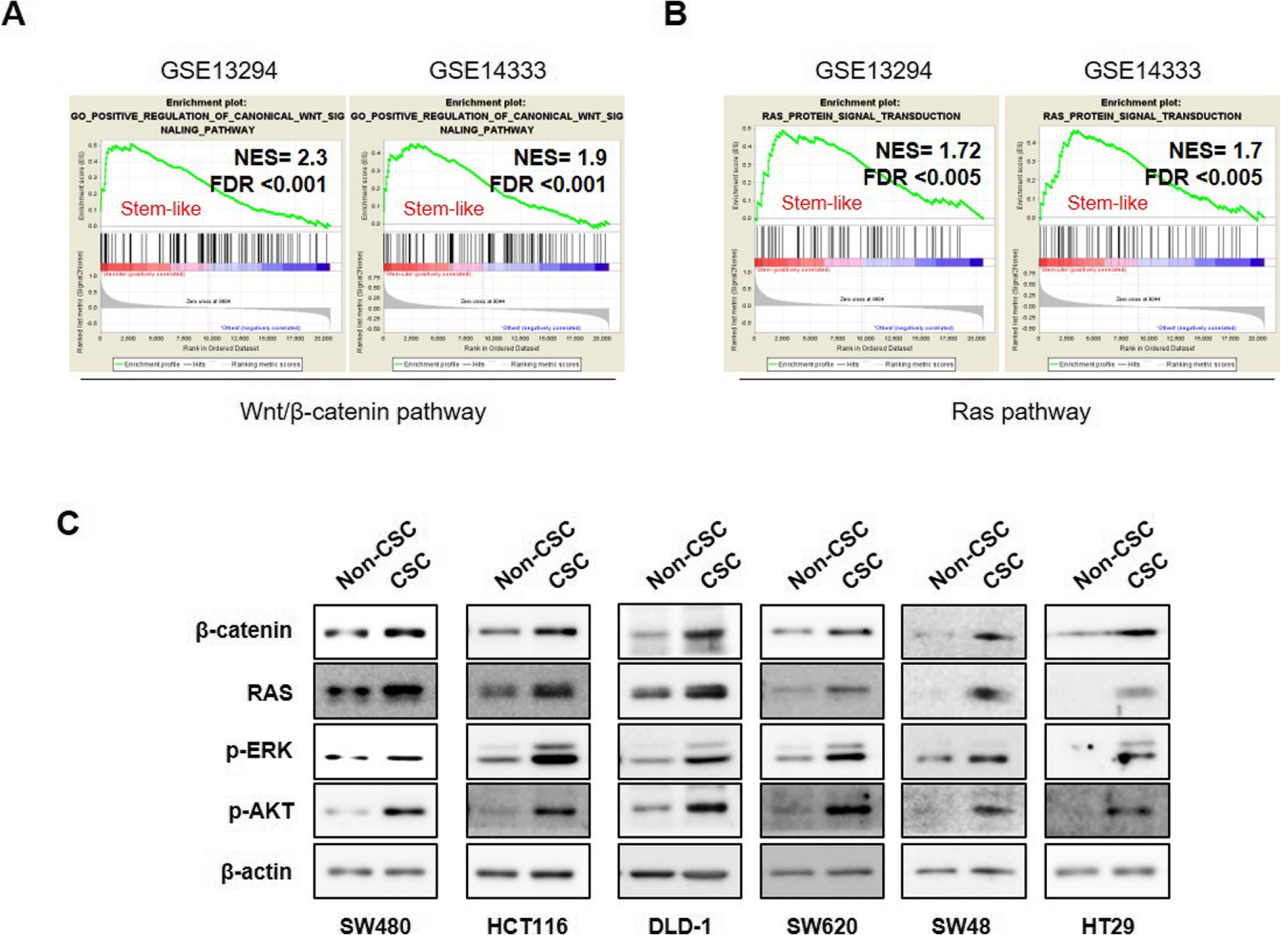
Both Wnt/β-catenin and RAS signaling pathways are activated in CRC harboring CSC properties. **a**, **b** GSEA of CRC patient tissues (GSE13294, *N* = 125; GSE14333, *N* = 246) of the (**a**) canonical Wnt and (**b**) RAS pathways in the stem-like subtype of CRC tumors (GSE13294, *N* = 20; GSE14333, *N* = 55) and other differentiated CRC tumors. **c** Immunoblot analyses of CRC cell lines comparing CSC-like (CSC) and non-CSC-like (non-CSC) populations of CRC cell lines using the indicated antibodies. CSC and non-CSC-like population of CRC cells were sorted by FACS using CD44, CD133, and CD166 antibodies

Due to the frequent mutations in the components of Wnt/β-catenin and RAS pathways in CRC and their critical roles in the synergistic activation of CSCs [15, 16, 24], we first investigated whether activation of these pathways are correlated with stem cell activities in the CRC patients. Gene set enrichment analysis (GSEA) using two differential gene expression profiles from CRC patient data sets (GSE13294; *N* = 125 and GSE14333; *N* = 246) showed that genes involved in the Wnt/β-catenin and RAS pathways are simultaneously elevated in stem-like subtype of CRC patient tumors that highly express stem cell-related gene signatures (GSE13294; *N* = 20 and GSE14333; *N* = 55) [7] compared with other differentiated CRC patient tumors (Fig. 1a, b). Since simultaneous activations of Wnt/β-catenin and RAS signaling pathways is associated with stabilizations of both β-catenin and RAS proteins by *APC* loss [18, 25], we examined the expression levels of β-catenin and RAS in the CSC-like populations which have higher spheroid forming sorted by FACS using CSC markers including CD44, CD133, and CD166 compared with those in the non-CSC populations. In addition, the CSC characteristic of CSC-like population was confirmed by their higher spheroid forming ability compared with non-CSC-population [23] (Additional file 1: Figure S1). β-catenin and RAS levels were highly elevated with activations of RAS downstream ERK and AKT kinases in CSC-like populations of CRC cell lines with various genetic backgrounds as shown by immunoblot analyses (Fig. 1c). Moreover, significant increases in the GTP-RAS, the active form of RAS, were observed in the CSC-like populations (Additional file 1: Figure S2), showing that the enriched RAS proteins in the CSC-like populations are active. These results suggest that β-catenin and RAS proteins would be effective molecular targets for suppressing the colorectal CSCs.

### The stem cell characteristics of CRC cell lines are suppressed by KYA1797K, the small molecule destabilizing both β-catenin and RAS

**Fig. 2:**
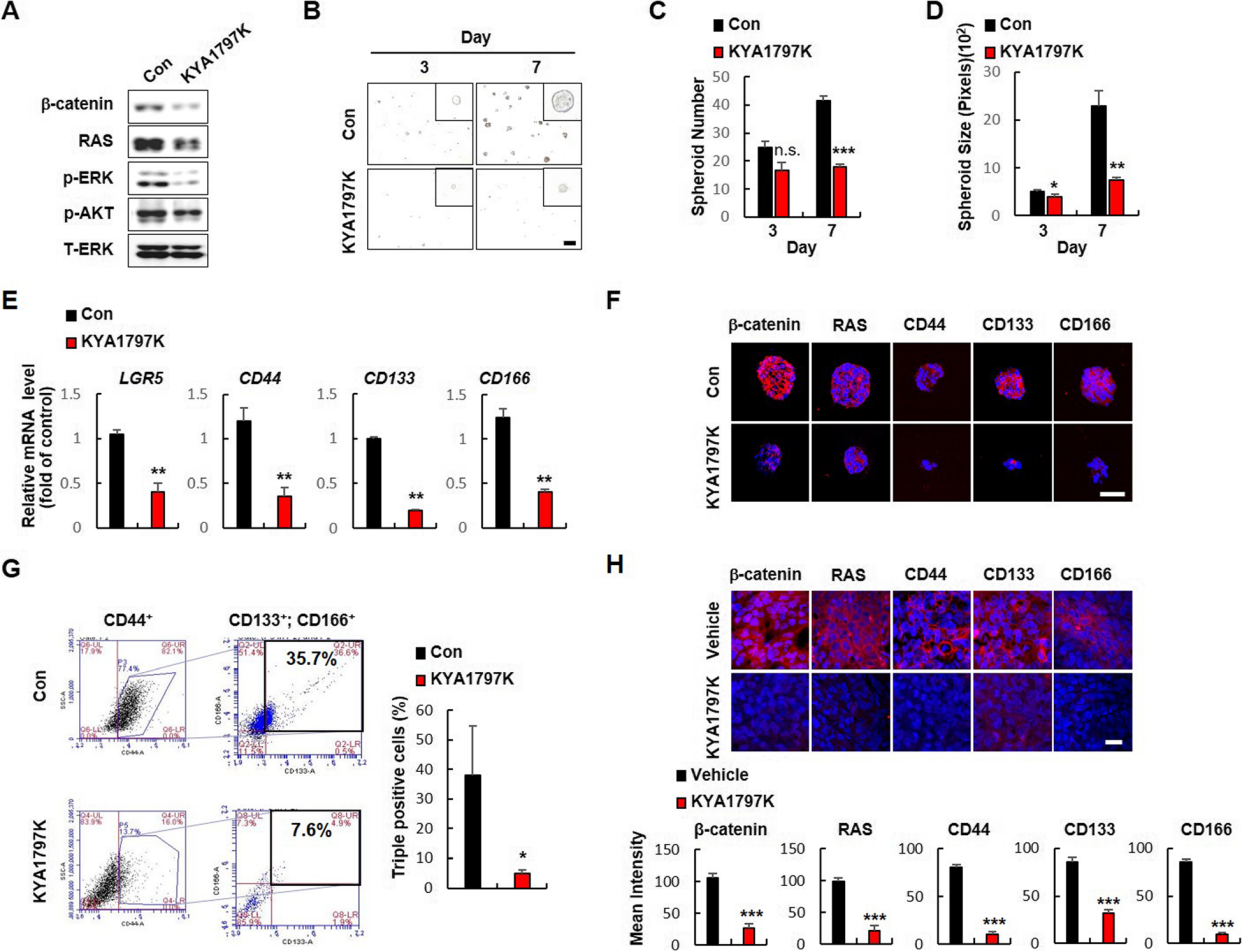
Effects of KYA1797K on the CSC properties of CRC cells harboring *APC* and *KRAS* mutations. DLD-*KRAS-*MT cells were seeded at a density of 1 × 10^4^ cells/mL in ultra-low attachment plates for spheroid culture and treated with DMSO or KYA1797K (25 μM). **a** Immunoblot analyses of DMSO- or KYA1797K-treated spheroids using the indicated antibodies. **b** Representative images of spheroids with DMSO or KYA1797K treatment on days 3 and 7 (*N* = 3). Scale bar represents 200 μm. **c**, **d** The numbers (**c**) and sizes (**d**) of spheroids were measured using Image J v1.47 software. **e** Relative mRNA levels of the CSC markers *LGR5*, *CD44*, *CD133,* and *CD166* in spheroids treated with DMSO or KYA1797K. **f** ICC analysis of spheroids treated with DMSO or KYA1797K using the indicated antibodies. Scale bar represents 50 μm. **g** FACS analyses of CD44, CD133, and CD166 triple-positive cells in spheroids treated with DMSO or KYA1797K. **h** Immunofluorescent IHC analyses of tumors from DLD-*KRAS-*MT xenograft mice treated with vehicle or KYA1797K using the indicated antibodies. Scale bar represents 20 μm. Quantification of fluorescence signals were performed using Zen software. In (**c**), (**d**), (**e**), (**g**), and (**h**), results are presented as mean ± SD of three independent experiments. *n.s*. no significance, * *P* < 0.05, ** *P* < 0.01, *** *P* < 0.001

Given that CSC-like populations of CRC cells have elevated protein levels of β-catenin and RAS, compared with non-CSC populations, we investigated whether the stem cell characteristics of CRC are associated with stabilization of β-catenin and RAS by treating the β-catenin and RAS destabilizing compound, KYA1797K [19], on the spheroid-cultured DLD-*KRAS-*MT CRC cells harboring both *APC* and *KRAS* mutations [16, 20]. KYA1797K effectively degraded both β-catenin, RAS, and inhibited the ERK and AKT activities (Fig. 2a) with decreased spheroid forming ability as measured by the numbers and sizes of spheroids (Fig. 2b- d). Consistent with the effects on sphere-forming ability, KYA1797K significantly reduced the mRNA levels of the CSC markers *LGR5*, *CD44*, *CD133* and *CD166* (Fig. 2e). Immunocytochemical analyses confirmed the KYA1797K-mediated decrease of β-catenin and RAS levels were followed by reduction of CSC markers in the DLD-*KRAS-*MT spheroids (Fig. 2f). Flow cytometry analyses also revealed that CSC populations having CD44^+^ CD133^+^ CD166^+^ triple positive cells were significantly decreased by KYA1797K (Fig. 2g; Additional file 1: Table S1). Consistently, similar inhibitory effects of KYA1797K on the CSC properties were also confirmed in SW480 and SW620 CRC cell lines (Additional file 1: Fig. S3 A-D). Moreover, immunohistochemistry (IHC) analyses of tumor tissues of DLD-1-*KRAS*-MT xenograft mice also revealed that KYA1797K effectively suppressed the cancer stemness of DLD-1-*KRAS*-MT cells (Fig. 2h).

### The small molecule-induced destabilization of both β-catenin and RAS suppresses the stemness of small intestinal tumors of *Apc*^*Min/+*^*/Kras*^*G12D*^*LA2* mice

**Fig. 3:**
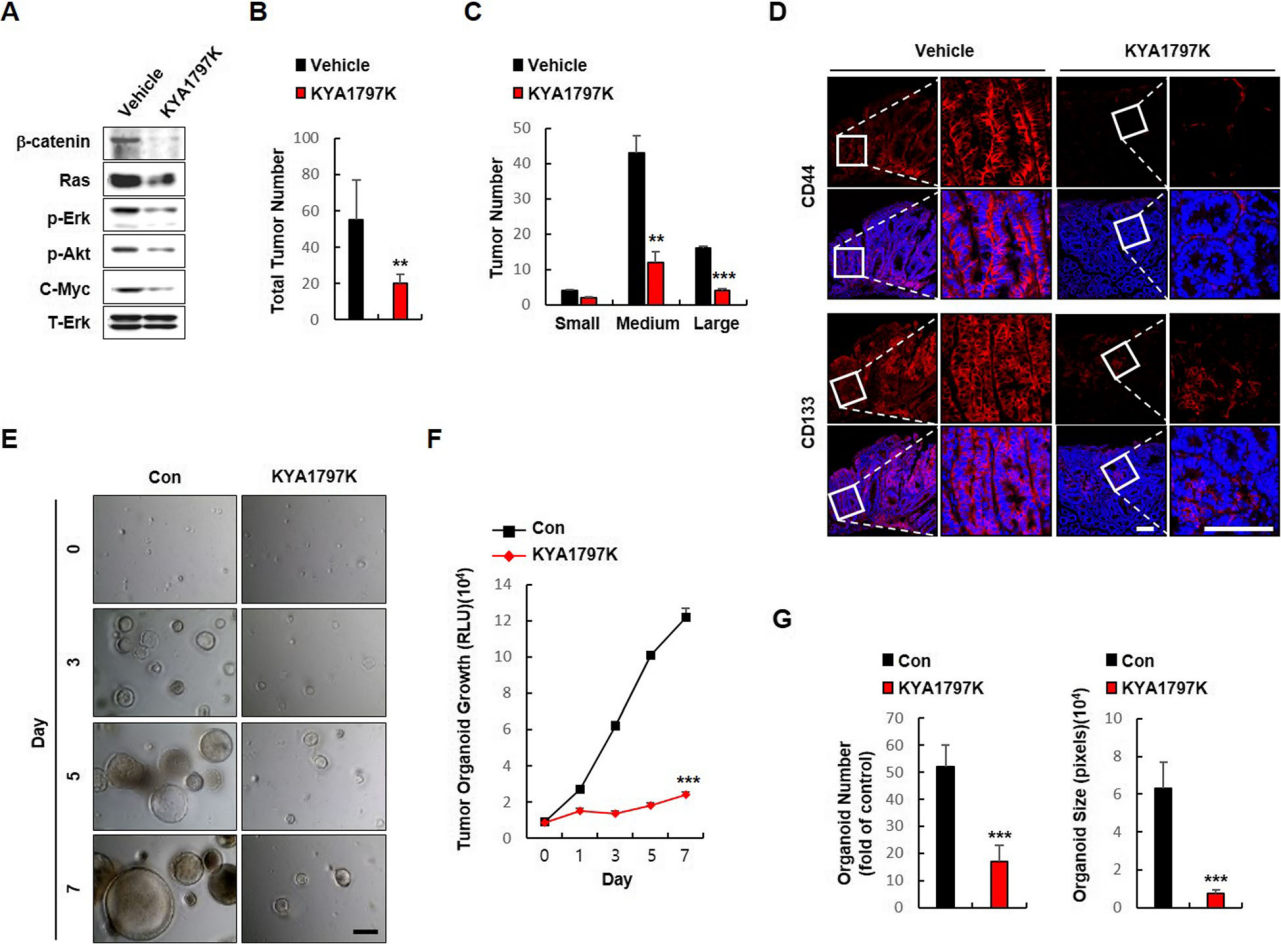
Effects of KYA1797K on the CSC properties of mice harboring *Apc* and *Kras* mutations. Analysis of 5-week-old *Apc*^*Min/+*^*/K-ras*^*G12D*^*LA2* mice treated with intraperitoneal (i.p.) injections of vehicle or KYA1797K (25 mg/kg) 5 days per week for 7 weeks (*N* = 4). **a** Immunoblots of vehicle- or KYA1797K-treated small intestinal tumors of *Apc*^*Min/+*^*/K-ras*^*G12D*^*LA2* mice using the indicated antibodies. **b, c** The numbers (**b**) and sizes (**c**) of vehicle- or KYA1797K-treated small intestinal tumors of *Apc*^*Min/+*^*/K-ras*^*G12D*^*LA2* mice. **d** IHC analyses of small intestinal tumors treated with vehicle or KYA1797K using the indicated antibodies. Representative images were captured using Zeiss confocal microscope from at least three different fields. Boxes indicate enlarged areas. Scale bar in the low-magnification images represents 20 μm, and the scale bar in the high-magnification images represent 100 μm. **e** Bright-field microscopy images of DMSO- or KYA1797K-treated tumor organoids derived from small intestinal tumor cells after 0, 3, 5, and 7 days. Representative images were captured using a Nikon 2000 U microscope from three different fields. **f** Viable cells were detected by luminescence production using CellTiter-Glo® Luminescent Cell Viability Assay. **g** Numbers and sizes of tumor organoids were measured using Image J v1.47 software. The quantitative data are presented as mean ± standard deviation. ** *P* < 0.01, *** *P* < 0.001

We examine whether KYA1797K effectively inhibit the stemness of CSCs in small intestinal tumors of *Apc*^*Min/+*^*/Kras*^*G12D*^*LA2* mice that exhibit synergistic activation of CSCs by *Apc* and *Kras* mutations (Additional file 1: Figure S4) [15, 16], we tested the effects of KYA1797K on the stemness of small intestinal tumors in *Apc*^*Min/+*^*/Kras*^*G12D*^*LA2* mice. KYA1797K treatment decreased the levels of β-catenin and Ras proteins as well as the activities of Erk and Akt in the small intestinal tumors of the *Apc*^*Min/+*^*/K-ras*^*G12D*^*LA2* mice reducing both of their number and size of tumors (Fig. 3a-c). IHC analyses revealed that KYA1797K treatment significantly reduced expression levels of CD44 and CD133 (Fig. 3d) as well as the convincing intestinal CSC markers, Lrig1 and EPHB3 (Additional file 1: Figure S6). Furthermore, the growth of tumor organoids derived from small intestinal tumor cells of *Apc*^*Min/+*^*/Kras*^*G12D*^*LA2* mice was effectively suppressed by KYA1797K treatment (Fig. 3e, f) along with significant decreases in the number and size of the tumor organoids at day 7 in the presence of EGF, R-spondin1, and Noggin, mimicking the in vivo condition of hyper-activated CSCs. (Additional file 1: Figure S5; Fig. 3g).

### KYA1797K suppresses the stemness of CSC in the CRC patient tumor-derived cells (PDCs) and patient-derived tumor xenograft (PDTX)

**Fig. 4:**
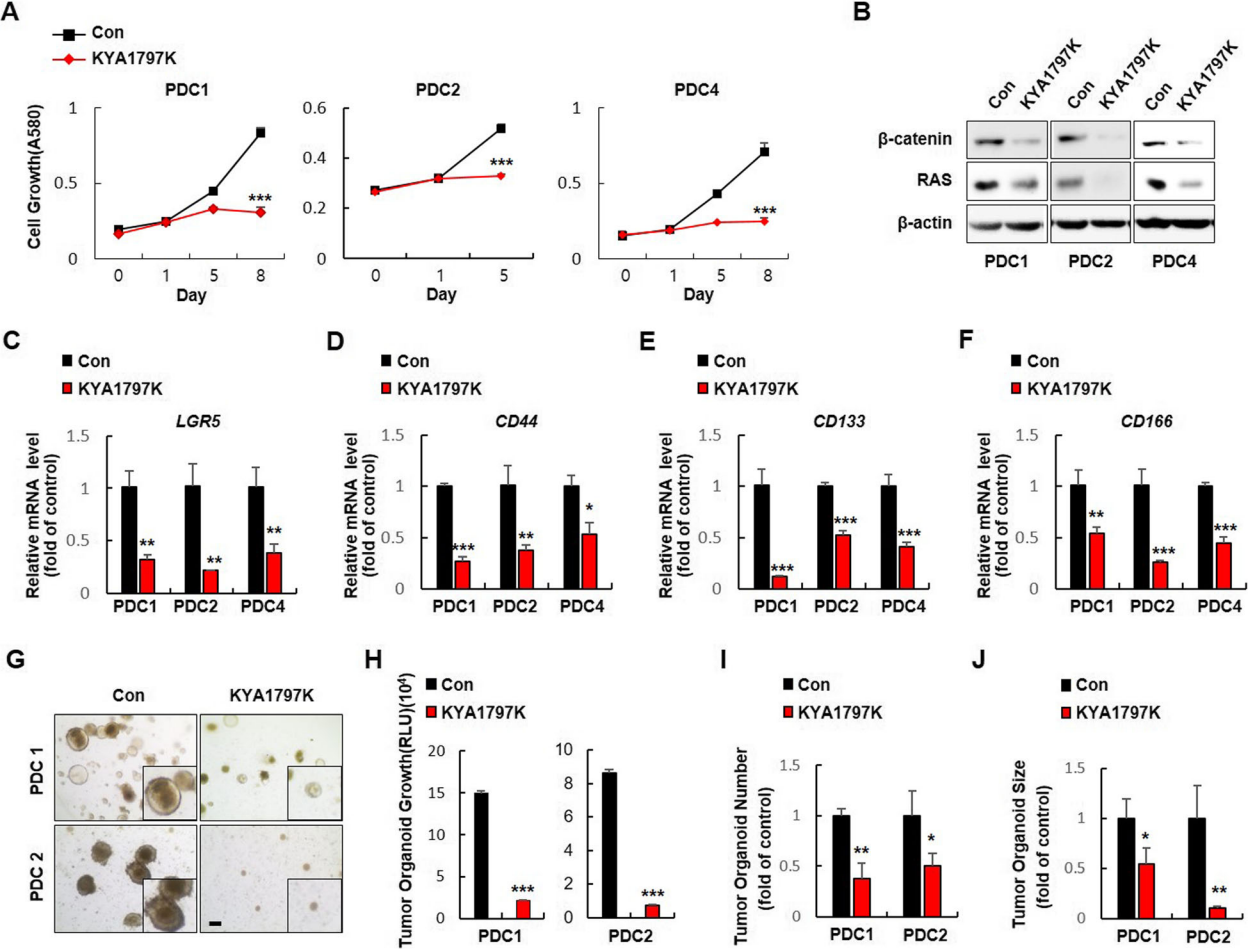
Effects of KYA1797K on the CSC potential in CRC PDCs. **a** Immunoblot analyses of PDCs treated with DMSO or KYA1797K (25 μM) were performed using the indicated antibodies. **b** The effect of KYA1797K on the growth of CRC PDC1, PDC2, and PDC4 were performed using a 3-(4,5-dimethylthiazol-2-yl)-2,5-diphenyltetrazolium bromide (MTT) assay. **c- f** qRT-PCR analyses of (**c**) *LGR5*, (**d**) *CD44*, (**e**) *CD133*, and (**f**) *CD166* were performed on KYA1797K- or DMSO-treated PDC1, PDC2, and PDC4. **g** The effects of KYA1797K on PDC1- and PDC2-derived tumor organoid formation (*N* = 3). **h** Viable cells were detected by luminescence production using the CellTiter-Glo Luminescent Cell Viability Assay. **i** Numbers and **j** sizes of PDC-derived tumor organoid formation were measured using Image J v1.47 software. The quantitative data are presented as mean ± standard deviation. * *P* < 0.05, ** *P* < 0.005, *** *P* < 0.001

To further explore the clinical relevance of destabilization of β-catenin and RAS in suppression of CSCs, we treated KYA1797K to CRC PDCs which detected mutation profiles of genes frequently occurred in CRC patients (Additional file 1: Table S2) [21] Treatment with KYA1797K significantly suppressed growth of PDCs (Fig. 4a) with reduction of β-catenin and RAS levels (Fig. 4b). In addition, KYA1797K effectively reduced the expression of CSC markers in PDCs (Fig. 4c-f). The inhibitory effects of KYA1797K on the formation of tumor organoids derived from PDC1 and PDC2 further confirmed the suppression of CSC characteristics (Fig. 4g- j).

**Fig. 5:**
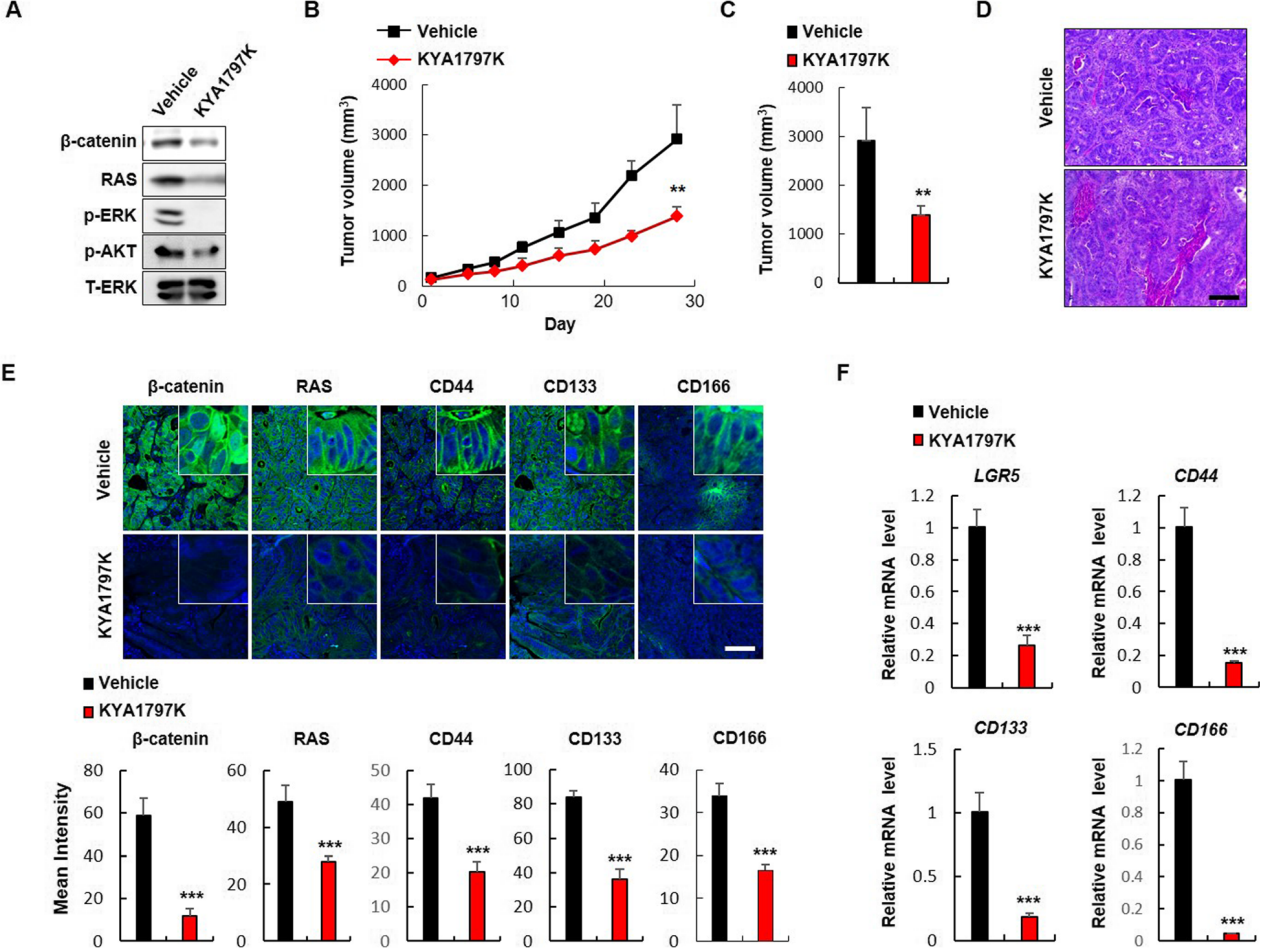
Effects of KYA1797K on the tumorigenic potential and stemness of CRC PDTX. NOD/SCID mice bearing CRC patient tumors were treated with vehicle or KYA1797K (25 mg/kg) via i.p. injection for 28 days (*N* = 5). **a** Immunoblot analyses of vehicle or KYA1797K treated PDTX using indicated antibodies. **b** Volumes of subcutaneous tumors treated with vehicle or KYA1797K were measured using calipers two times a week for 28 days. **c** Final tumor volumes of vehicle- or KYA1797K-treated CRC PDTX. **d** Hematoxylin and eosin staining of vehicle- or KYA1797K-treated PDTX. Scale bar represents 200 μm. **e** IHC analyses of vehicle- or KYA1797K-treated PDTX using the indicated antibodies. Scale bar represents 100 μm. Mean intensities of fluorescence of indicated markers were quantified using Zen software in at least three different tissue samples. **f** qRT-PCR analyses of *LGR5*, *CD44*, *CD133*, and *CD166* mRNAs in vehicle- or KYA1797K-treated CRC PDTX samples. Quantitative data are presented as mean ± standard deviation. * *P* < 0.05, ** *P* < 0.005, *** *P* < 0.001

To investigate the effects of destabilization of β-catenin and RAS on CSC activation related to metastasis, we subcutaneously implanted NOD/SCID mice with *KRAS* mutated colon adenocarcinoma metastasized to lung, and measured effect of KYA1797K treatment. KYA1797K decreased levels of both β-catenin and RAS with inactivation of ERK and AKT kinases in the tumor lysates of the PDTX (Fig. 5a). The growth of tumors was effectively inhibited during time course up to 28 days (Fig. 5b) and tumor volumes were reduced to 58% compared to vehicle treated tumors at 28 days after KYA1797K treatment (Fig. 5c). Hematoxylin and Eosin staining analyses showed that PDTX tissues derived from colon adenocarcinoma metastasized to lung still retain the colon adenocarcinoma histology and their histology was not altered by KYA1797K treatment (Fig. 5d). Furthermore, KYA1797K effectively suppressed the expressions of CSC markers with decreased levels of β-catenin and RAS (Fig. 5e, f), while it significantly increased the expression of CK20, a convincing differentiation marker, in PDTX tissues (Additional file 1: Figure S7), suggesting that KYA1797K suppresses the stemness of CRC stem cells.

## Discussion

Aberrant activation of the Wnt/β-catenin and RAS signaling pathways with elevated β-catenin and RAS protein levels has been observed in various human cancers including CRC, gastric cancer, endometrium cancer, and non-small cell lung cancer (NSCLC) [19, 26–30]. Therefore, small molecule-mediated destabilization of both β-catenin and RAS has been shown to suppress the tumor growth in cancers such as CRC, gastric cancer, and NSCLC The effectiveness of small molecules to many different types of cancers is due to the destabilization of β-catenin and RAS proteins which are overexpressed in the different types of cancer [19, 28–30], suggesting that stabilization of β-catenin and RAS play critical roles in regulating the activation of Wnt/β-catenin and RAS signaling pathways in many types of cancers as well as the effect of their component gene mutation.

In CRCs, both β-catenin and RAS protein levels are highly elevated mainly due to *APC* mutations occurring in as many as 90% of CRC patients [18, 19, 25]. In the presence of *APC* mutation, additional *KRAS* mutation synergistically activates the Wnt/β-catenin signaling via a positive feedback loop through the MEK-ERK pathway, resulting in activation of CSCs [16]. In this study, we observed that Wnt/β-catenin and RAS signaling pathways are highly activated in the stem-like subtype of CRCs that possess CSC characteristics. Of interest, β-catenin and RAS protein levels as well as the active form of RAS (GTP-RAS) are significantly higher in CD44, CD133, and CD166 triple-high populations of CRC cells which possess the higher spheroid forming ability, compared with their low counterparts, suggesting that suppression of the Wnt/β-catenin and RAS pathways, especially by reduction of the levels of β-catenin and RAS could be an effective approach to control CSCs in CRC and have led us to investigate the effects of the β-catenin and RAS destabilizing compound KYA1797K on inhibition of CRC stem cells. Destabilization of β-catenin and RAS by KYA1797K effectively suppressed the stemness of colorectal CSCs as confirmed by suppression of the CSC markers including LGR5, CD44, CD133, and CD166 with significant inhibition of Wnt/β-catenin and RAS pathways in various spheroid cultured CRC cell lines. The suppressive effects of KYA1797K on the stemness of small intestinal tumors were also confirmed in *Apc*^*Min/+*^*/Kras*^*G12D*^*LA2* mouse model and its small intestinal tumor cell-derived tumor organoids in the presence of EGF, Noggin, and R-spondin-1 which mimics the hyper-activation conditions of LGR5^+^ CSCs in vivo. Moreover, the clinical relevance of the destabilization of β-catenin and RAS by KYA1797K on the suppression of CSCs was validated by using the patient avatar models such as CRC PDCs and PDTX derived from CRC patient tissues that had metastasized to lung. Especially, considering the importance of CSCs in the development of metastasis [16], the inhibitory effects of KYA1797K on the growth of lung-metastasized colon cancer PDTX were confirmed with significant suppression of CSC properties and induction of differentiation, suggesting that our approach for simultaneous degradation of both β-catenin and RAS is a potential approach for suppression of metastasis as well as the stemness of CRC cells.

## Conclusion

we demonstrated that small molecule-mediated destabilization of β-catenin and RAS offer a potential approach to control CSCs in CRC. Considering the importance of CSC activation in tumor metastasis and recurrence and its association with poor patient survival and drug resistance, this therapeutic approach to suppress the CSCs would be an effective strategy for improving clinical outcomes in CRC patients.

## Supplementary information

**Additional file 1: Figure S1** The scheme for classifying CSC and non-CSC populations from CRC cells. **Figure S2**. GTP-loaded active RAS levels are highly elevated with the levels of RAS in CSC-like CRC cells compared with non-CSC cells. **Figure S3**. Effects of KYA1797K on CSC properties of CRC cells harboring *APC* and *KRAS* mutations. **Figure S4**. *Apc* and *Kras* mutations synergistically activate the CSC properties in murine small intestinal tumor cells. FACS analyses of CD44 and CD133 double-positive cells. **Figure S5**. LGR5^+^ CSCs intermingled with paneth cells which secrete EGF, R-spondin1, and Noggin were more activated than LGR5^+^ CSCs not intermingled with paneth cells. **Figure S6**. Effect of KYA1797K on suppression of CSC properties in small intestinal tumors of *Apc*^*Min/+*^*/ K-ras*^*LA2*^. **Figure S7**. KYA1797K significantly induces the KRT20 in CRC PDTX. **Table S1**. Effects of KYA1797K on CSC populations of CRC cells. Alteration of CSC marker positive cells by KYA1797K treatment were analyzed by flow cytometry. **Table S2**. Genetic profiles of PDC. The mutation status of *APC*, *PIK3CA*, *KRAS*, *EGFR*, *PI3K*, and TP53 in PDCs

## Data Availability

All data in our study are available upon request.

## Abbreviations

APC: Adenomatous polyposis coli
CRC: Colorectal cancer
CSC: Cancer stem cell
DFS: Disease free survival
ERK: Extracellular signal-regulated kinase
ISC: Intestinal stem cells
LGR5: Leucine-rich repeat-containing G-protein coupled receptor 5
PDC: Patient derived cell
PDTX: Patient derived tumor xenograft
PI3K: Phosphatidyl inositol 3-kinase
