# Supplementary material for: Small molecule-induced simultaneous destabilization of β-catenin and RAS is an effective molecular strategy to suppress stemness of colorectal cancer cells
Source: Cell Commun Signal. 2020 Mar 6;18:38. doi: 10.1186/s12964-020-0519-z (PMC7060567; doi:10.1186/s12964-020-0519-z)
Supplement: Supplementary file 2 — Additional file 1: Figure S1 The scheme for classifying CSC and non-CSC populations from CRC cells. Figure S2. GTP-loaded active RAS levels are highly elevated with the levels of RAS in CSC-like CRC cells compared with non-CSC cells. Figure S3. Effects of KYA1797K on CSC properties of CRC cells harboring APC and KRAS mutations. Figure S4. Apc and Kras mutations synergistically activate the CSC properties in murine small intestinal tumor cells. FACS analyses of CD44 and CD133 double-positive cells. Figure S5. LGR5+ CSCs intermingled with paneth cells which secrete EGF, R-spondin1, and Noggin were more activated than LGR5+ CSCs not intermingled with paneth cells. Figure S6. Effect of KYA1797K on suppression of CSC properties in small intestinal tumors of ApcMin/+/ K-rasLA2. Figure S7. KYA1797K significantly induces the KRT20 in CRC PDTX. Table S1. Effects of KYA1797K on CSC populations of CRC cells. Alteration of CSC marker positive cells by KYA1797K treatment were analyzed by flow cytometry. Table S2. Genetic profiles of PDC. The mutation status of APC, PIK3CA, KRAS, EGFR, PI3K, and TP53 in PDCs [file 12964_2020_519_MOESM1_ESM.docx]

**Small molecule-induced simultaneous destabilization of β-catenin and RAS is an effective molecular strategy to suppress stemness of colorectal cancer cells**

Yong-Hee Cho^1,2^, Eun Ji Ro^1,2^, Jeong-Su Yoon^1,2^, Dong–Kyu Kwak^1,2^, Dong-Woo Kang^4^, Kang-Yell Choi^1,2, 3^

^1^Translational Research Center for Protein Function Control, Yonsei University, Seoul, Korea. ^2^Department of Biotechnology, College of Life Science and Biotechnology, Yonsei University, Seoul, Korea.

^3^CK Biotechnology Inc., Building 117, 50 Yonsei Ro, Seodaemun-Gu, Seoul, Korea

^4^Medpacto Inc., Borim building, 92 myeongdal Ro, Seocho-gu, seoul, Korea.

**Corresponding author**: Kang-Yell Choi, ^1^Translational Research Center for Protein Function Control, ^2^Department of Biotechnology, College of Life Science and Biotechnology, Yonsei University, Seoul, Korea. ^3^CK Biotechnology Inc., Building 117, 50 Yonsei Ro, Seodaemun-Gu, Seoul, Korea. Tel.: +82-2-2123-2887; Fax: +82-2-2123-8284

**Supplementary Figures and Legends**

**
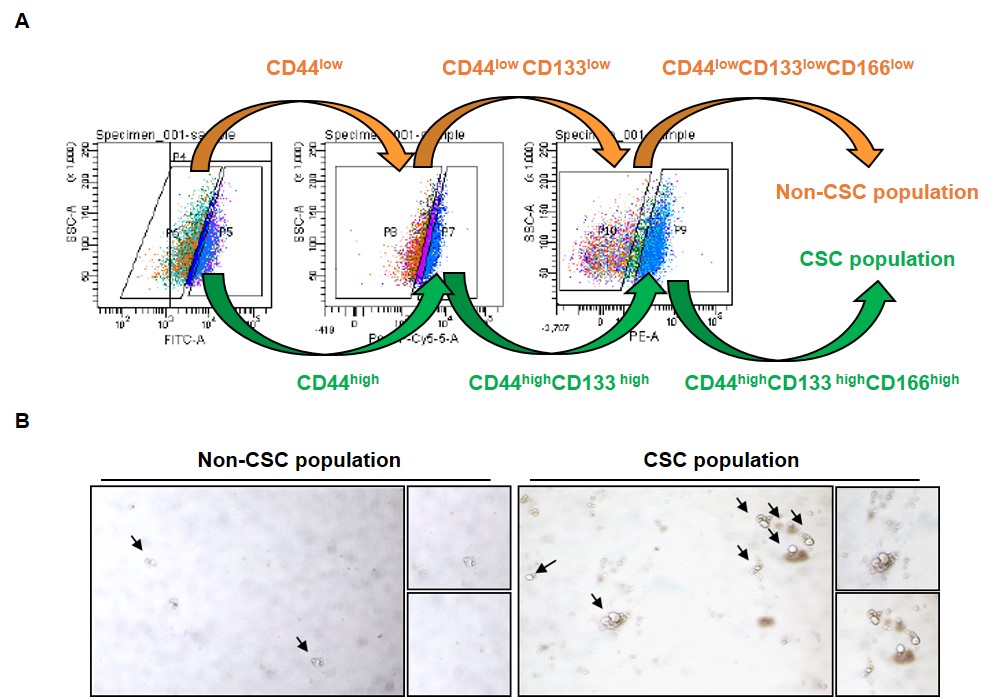
**

**Figure S1. The scheme for classifying CSC and non-CSC populations from CRC cells. (A)** Fluorescence activated cell sorting (FACS) analyses of CRC cells using CD44, CD133, and CD166 antibodies. The CSC population consists of cells expressing the CD44^high^, CD133^high^, CD166^high^ and non-CSC population consists of cells expressing CD44^low^, CD133^low^, CD166^low^. (**B**) CSC and Non-CSC populations of D-MT cells sorted by FACs by using CD44, CD133, and CD166 antibodies were seeded at a density of 1 x 10^3^ cells in ultra-low attachment 96 well plates for 5 days.

**
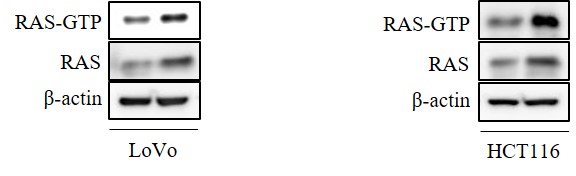
**

**Figure S2. GTP-loaded active RAS levels are highly elevated in CSC-like CRC cells in accordance with the high levels of RAS compared with non-CSC cells.** CSC and non-CSC populations of SW480 and HCT116 CRC cells were classified by FACs using CD44, CD133, and CD166 antibodies. GTP-loaded active RAS with GST fusion protein in corresponding to the RAS binding domain of RAF (GST-RAF-RBD) evaluated RAS activation. Immunoblot analyses were performed using whole cell lysates with indicated antibodies.

**
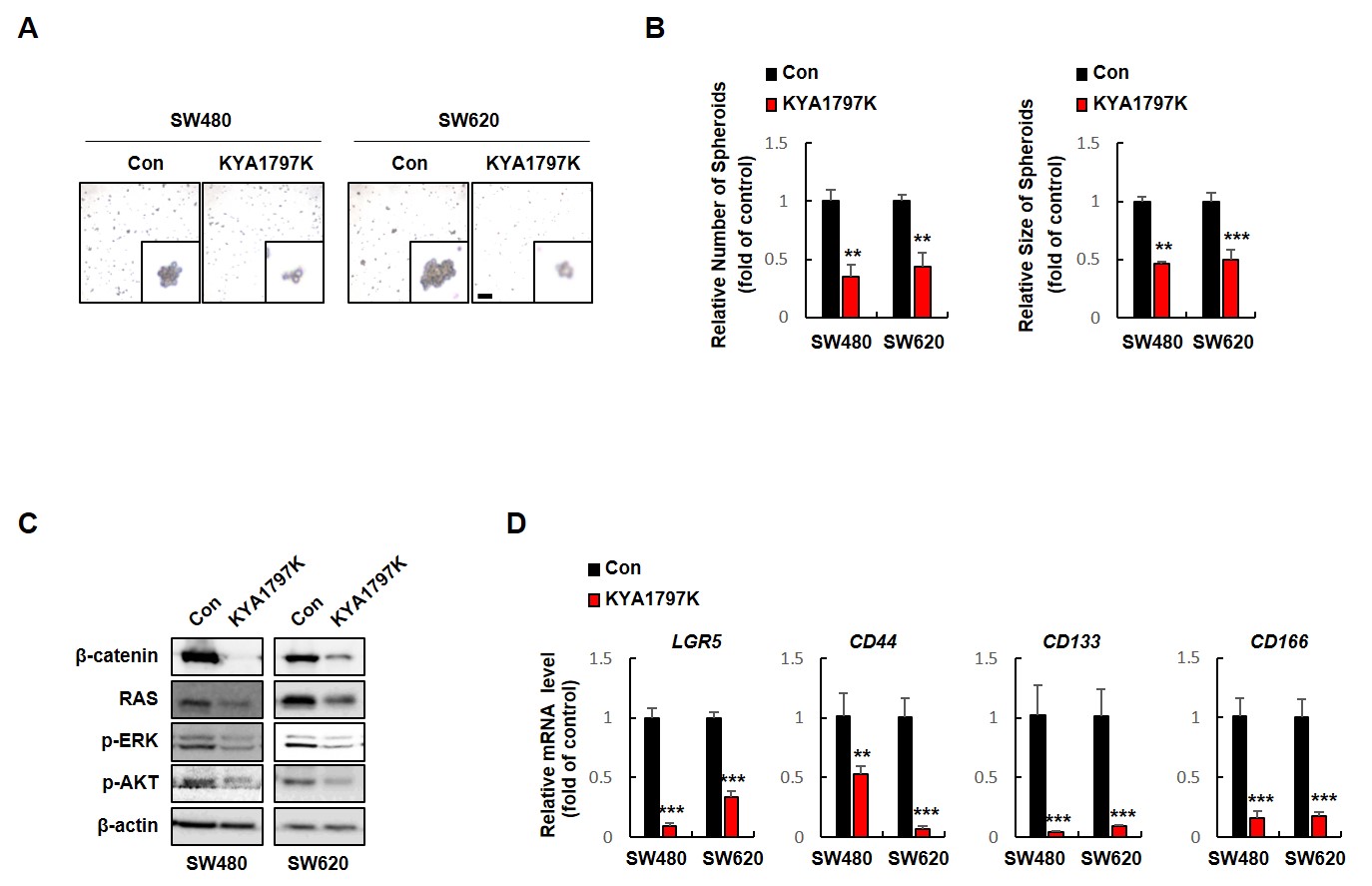
**

**Figure S3.** **Inhibitory effects of KYA1797K on the CSC properties of CRC cells harboring *APC* and *KRAS* mutations.** SW480 and SW620 cells were seeded at 1×10^4^ cells/mL in ultra-low attachment plates for spheroid culture and treated with DMSO or KYA1797K (25 μM). (**A**) Representative images of SW480 and SW620 spheroids treated with DMSO or KYA1797K on day 7. Scale bar represents 200 μm. (**B**) The numbers and sizes of the SW480 and SW620 spheroids were measured using Image J v1.47 software (*N*=3). (**C**) Immunoblot analyses of DMSO- or KYA1797K-treated SW480 and SW620 spheroids using the indicated antibodies. (**D**) Relative mRNA levels of CSC markers *LGR5*, *CD44*, *CD133,* and *CD166* in spheroids treated with DMSO or KYA1797K. The quantitative data are presented as mean ± standard deviation. ** *P*<0.01, *** *P*<0.001

]


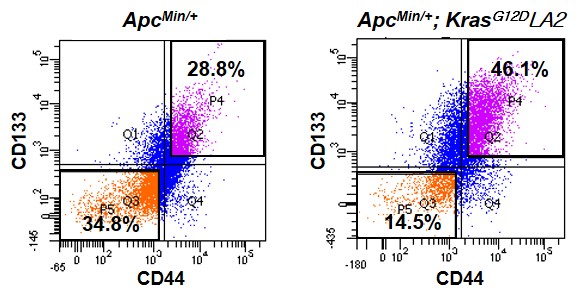


**Figure S4. *Apc* and *Kras* mutations synergistically activate the CSC properties in murine small intestinal tumor cells.** FACS analyses of CD44 and CD133 double-positive cells comparing small intestinal tumors of *Apc^Min/+^* and *Apc^Min/+/^Kras^G12D^LA2*


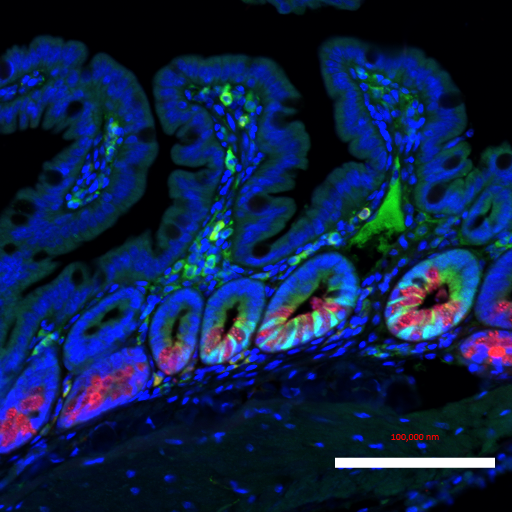

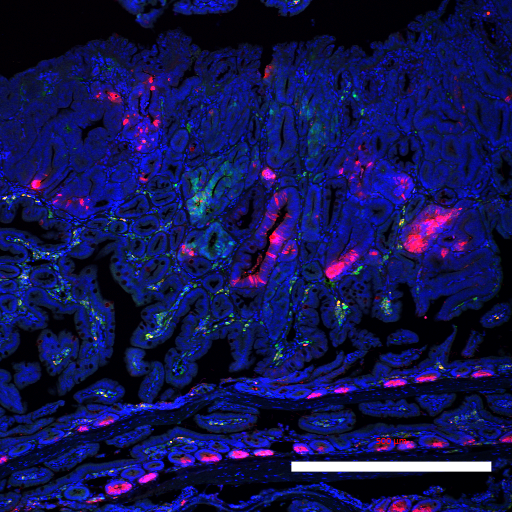

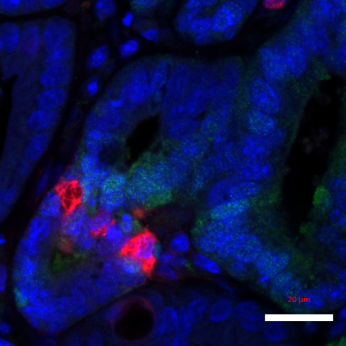

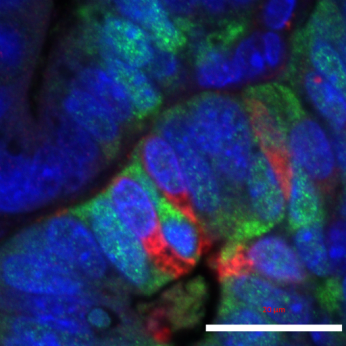

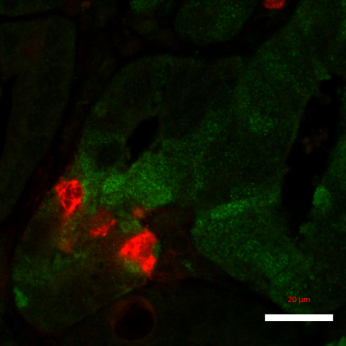

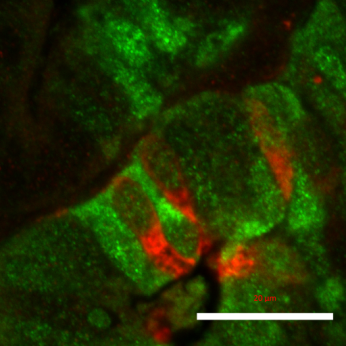


**LGR5 (EGFP)**

**Paneath cells (lysozyme)**

**Normal**

**Tumor**

***Apc^Min/+^/Lgr5^EGFP^***

***Apc^Min/+^/Lgr5 ^EGFP^***

**A**

**B**

**Figure S5.** LGR5^+^ CSCs intermingled with paneth cells which secrete EGF, R-spondin1, and Noggin were more activated than LGR5^+^ CSCs not intermingled with paneth cells**.**

(**A**) Immunohistochemical (IHC) analyses of small intestines of *Apc^Min/+^/Lgr5^EGFP^* using indicated antibodies. Scale bar represents 100 μm. (**B**) IHC analyses of small intestinal tumors of *Apc^Min/+^/Lgr5^EGFP^* using indicated antibodies. Scale bar represents 500 μm. Scale bars of enlarged images represent 20 μm.

**
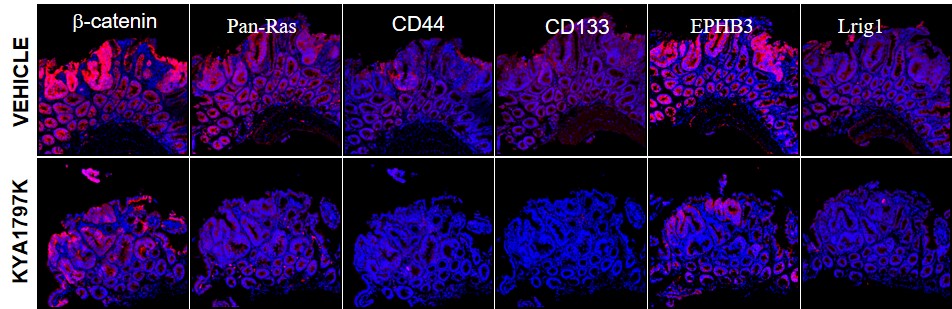
**

**Figure S6. KYA1797K effectively suppresses CSC properties in small intestinal tumors of *APC^Min/+^/ K-ras^LA2^***. IHC analyses of small intestinal tumors of *APC^Min/+^/K-ras^LA2^* mice, treated with intraperitoneal (i.p.) injections of vehicle or KYA1797K (25 mg/kg) 5 days per week for 7 weeks (*N*=4). Indicated antibodies were used in IHC.

**
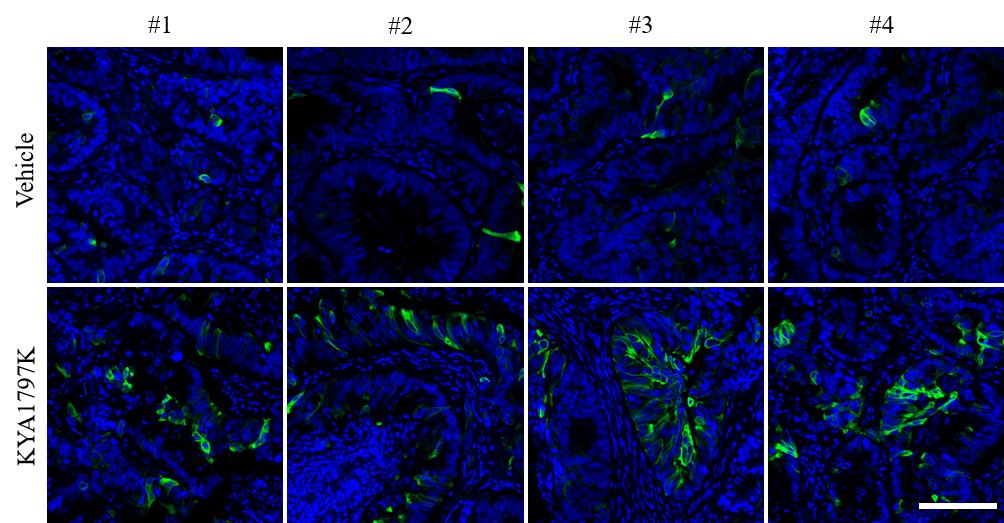
**

**Figure S7. KYA1797K significantly induces the KRT20 in CRC PDTX.** IHC analyses of vehicle- or KYA1797K-treated PDTX using the KRT20 antibody. Scale bar represents 100 μm


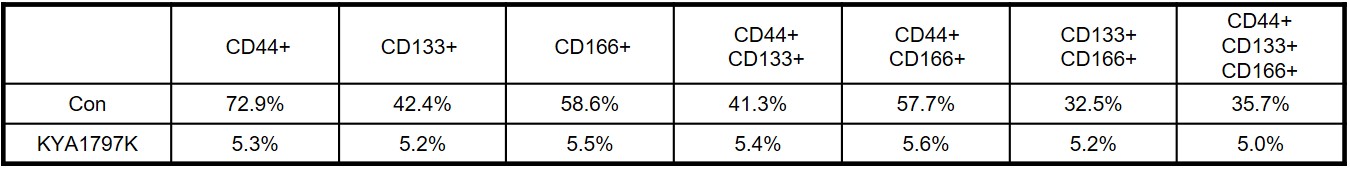


**Table S1. Effects of KYA1797K on CSC populations of CRC cells.** FACS analyses of CSC marker positive cells using CD44, CD133 cells in spheroids treated with DMSO or KYA1797K (25 μM).


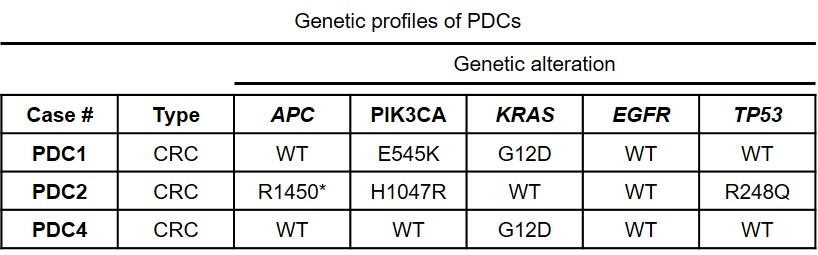


**Table S2.** Genetic profiles of CRC PDC. The mutation status of *APC*, *PIK3CA*, *KRAS*, *EGFR*, *PI3K*, and TP53 in PDCs
